# Dissection of partial 21q monosomy in different phenotypes: clinical and molecular characterization of five cases and review of the literature

**DOI:** 10.1186/s13039-016-0230-3

**Published:** 2016-02-24

**Authors:** Edoardo Errichiello, Francesca Novara, Anna Cremante, Annapia Verri, Jessica Galli, Elisa Fazzi, Daniela Bellotti, Laura Losa, Mariangela Cisternino, Orsetta Zuffardi

**Affiliations:** 1 Department of Molecular Medicine, University of Pavia, Via Forlanini 14, 27100 Pavia, Italy; 2 National Neurological Institute IRCCS C, Mondino, Pavia, Italy; 3 Mother-Child Department, Child Neurology and Psychiatry Unit, Spedali Civili, Brescia, Italy; 4 Department of Molecular and Translational Medicine, University of Brescia, Brescia, Italy; 5 Department of Pediatrics, IRCCS Policlinico San Matteo, University of Pavia, Pavia, Italy

**Keywords:** Array Comparative Genomic Hybridization (array-CGH), Behavioral disorders, BTG3 (BTG family, Member 3), DNA Copy Number Variations (CNVs), GRIK1 (glutamate receptor, Ionotropic, Kainate 1), Intellectual Disability (ID), Partial 21q monosomy, RBM11 (RNA binding motif protein 11)

## Abstract

**Background:**

Partial deletion of chromosome 21q is a very rare chromosomal abnormality associated with highly variable phenotypes, such as facial dysmorphic features, heart defects, seizures, psychomotor delay, and severe to mild intellectual disability, depending on the location and size of deletions. So far, three broad deletion regions of 21q have been correlated with the clinical phenotype.

**Results:**

We described the clinical and genetic features of three family members (father and two siblings) and other two unrelated patients with very wide range in age of diagnosis. All of them showed intellectual disability with very variable symptoms, from mild to severe, and carried 21q interstitial deletions with different sizes and position, as detected by conventional karyotype and array-CGH.

**Conclusions:**

Our study provided additional cases of partial 21q deletions, allowing to better delineate the genotype-phenotype correlations. In contrast to previous observations, we showed that deletions of the 21q proximal region are not necessarily associated with severe phenotypes and, therefore, that mild phenotypes are not exclusively related to distal deletions. To the best of our knowledge, this is the first report showing 21q deletions in adult patients associated with mild phenotypes, mainly consisting of neurobehavioral abnormalities, such as obsessive-compulsive disorders, poor social interactions and vulnerability to psychosis.

## Background

Partial deletion of chromosome 21q (ORPHA574) is a very rare condition (<1/1,000,000) associated with highly variable phenotypes, which include facial dysmorphic features, heart defects, seizures, psychomotor delay, and severe to mild intellectual disability, depending on the size and position of the deletion [1].

In one of the most complete studies to date, Lyle et al*.* [2] reported 11 cases of partial monosomy 21 and outlined three deletion regions with the associated phenotypic severity ranging from mild to severe to lethal. Deletions in the first region, ranging from the centromere to approximately 31.2 Mb (21q21.3), are associated with a severe phenotype. Deletions in the second region (31.2–36 Mb), corresponding to the 21q22.11 band with a higher gene density, produce a severe phenotype not compatible with survival. On the other hand, deletions in the third region, from 36 to 37.5 Mb to the telomere (21q22.12-qter, approximately 10 Mb), result in a relatively mild phenotype. However, other studies reported patients with proximal deletions of chromosome 21 and mild or even normal phenotypes [3–5].

In this study, we investigated five patients from three unrelated Italian families with deletions of chromosome 21q by conventional and molecular karyotyping (array-CGH), in order to underline new insights on genotype-phenotype correlations. In addition, previously published cases with chromosome 21q monosomies and similar deletions have been reviewed. Altogether, our data provide a further dissection of the complex 21q monosomy phenotype.

## Results

### Clinical reports

#### Patient 1

Patient 1 was a 53 years old male, father of cases 2 and 3. The patient was born full-term. He achieved the middle school graduation and then experienced different jobs (gardener, bricklayer, crane worker), being frequently fired after short periods. His mother (79 years old) was affected by hypertension and the father (80 years old) by insulin dependent diabetes mellitus and stroke. The patient had a 55 years old brother who was also affected by diabetes. He was first evaluated at the age of 53 years, after that chromosome 21 deletion had been ascertained in his children.

**Fig. 1:**
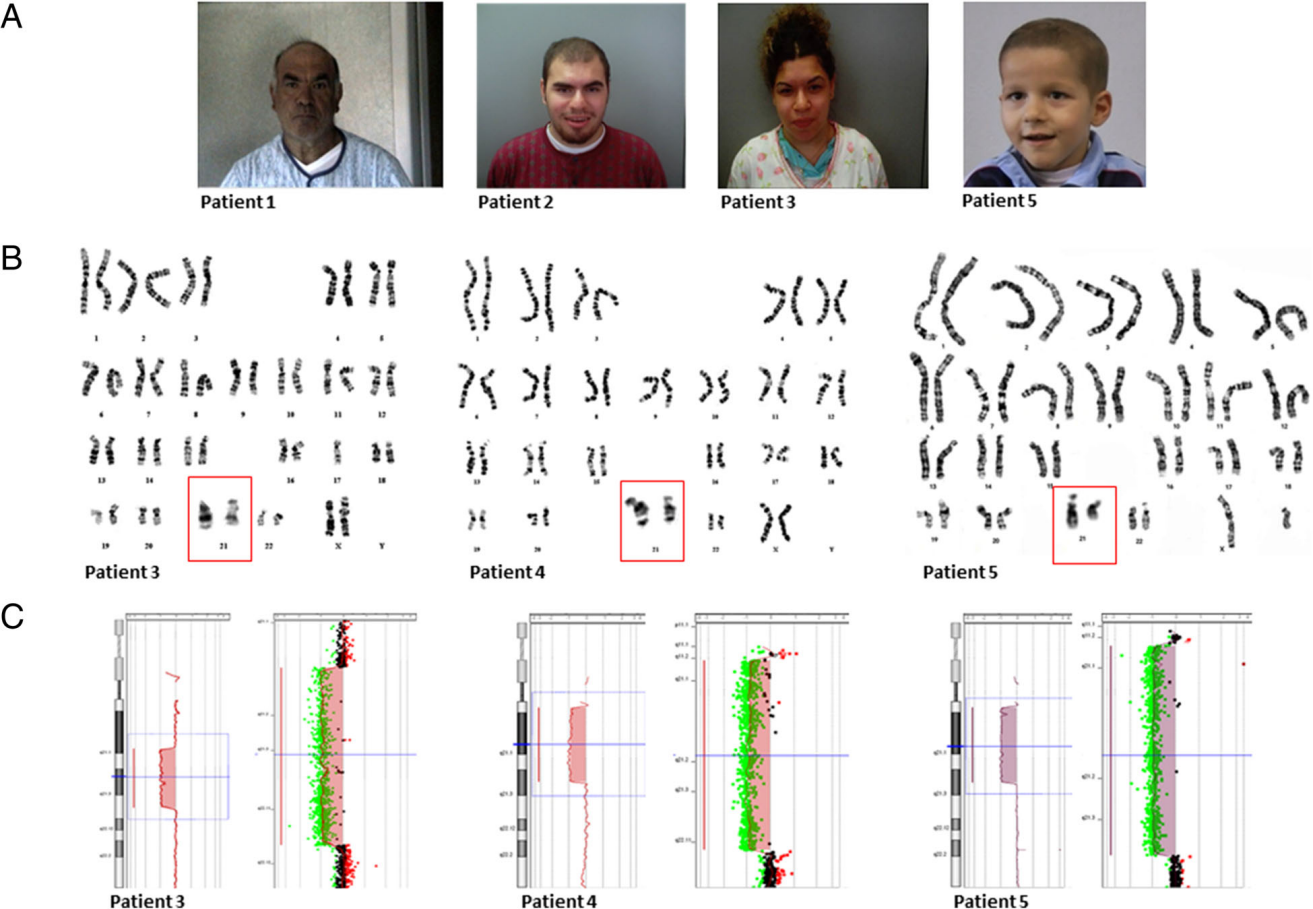
Clinical features (**a**), G-banded karyotypes (**b**) and array-CGH profiles (**c**) of patients with chromosome 21q deletions. Chromosomes 21 shown in the red boxes (**b**) are enlarged in respect to the original karyograms. Parents of patient 4 denied permission to publish pictures

The clinical evaluation documented mild facial dysmorphisms, such as deep set eyes, large ears and prominent nose (Fig. 1a). The neurological evaluation revealed mild head tremor and postural hand tremor. The Nuclear Magnetic Resonance (NMR) documented a periventricular white matter hyperintensity and enlarged cisterna magna. The electromyography (EMG) showed bilateral entrapment of median nerve at the wrist. The patient was scarcely collaborating and quite anxious. He frequently showed the tendency to give a very positive image of himself. The language was rather simple. The evaluation by DSM-IV (Diagnostic and Statistical Manual of Mental Disorders, Fourth Edition) documented a psychotic disorder with persecutory delusion. High levels of aggressiveness and impairment in behavioural control were well documented as well as alcohol abuse. Total IQ was 87, without any important discrepancy among verbal subtests (Verbal IQ = 88,) and the performance ones (Performance IQ = 88). Poor attentive skills, short-term memory and limited funds of knowledge acquired through school and cultural experience were documented by verbal subtests. Abstract thinking and understanding were very simple, with limited ability to synthesize verbal relationships and social knowledge. The computational skills were also very limited, as well as the logical-deductive and abstract reasoning.

#### Patient 2

Patient 2 was the oldest child of patient 1. He was born at term by caesarean section (weight 2,900 g). He started walking at 18 months and pronounced the first comprehensible words at 14 months. He attended 3 years of pre-school and started the primary school at 7 years. Then he attended a professional school care for mentally-disabled people. He was first evaluated at the age of 20 years because of mild intellectual disability, requiring a dedicated teacher at school, and autistic-like features.

The clinical examination revealed a coarse facies with nuchal low set hair, frontal alopecia, prominent forehead, small palpebral fissures, prominent nose, prognathism and large ears (Fig. 1a). The neurological evaluation revealed mild postural hand tremor and altered saccadic eye movements. The Magnetic Resonance Imaging (MRI), performed at the age of 18 years, showed mild periventricular white matter hyperintensity and enlarged cisterna magna. The EMG revealed mild sensorimotor axonal neuropathy and autonomic dysfunction. During examination, the patient showed adequate levels of attention and concentration. The evaluation by DSM-IV criteria for ID diagnosis documented the presence of a compulsive-obsessive disorder. The patient referred binge eating episodes, occurring during the night, and compulsive smoking (60 cigarettes/day). He was sufficiently autonomous and able to work. His global IQ was 79 (borderline level), without discrepancy among the verbal subtests (Verbal IQ = 79) and the performance ones (Performance IQ = 82). He demonstrated good comprehension skills, but low attitudes in numerical reasoning and a poor vocabulary. Selective and focused attention, mental control and response flexibility, as assessed by the Stroop Test, resulted inadequate.

#### Patient 3

Patient 3 was a 22 years old female, the younger child of patient 1. She was born at term with caesarean section (weight 2,600 g). She pronounced the first words at 18 months of age. She was first evaluated at the age of 18 years because of mild intellectual disability requiring a dedicated teacher at school.

The clinical evaluation revealed coarse facies with nuchal low set hair, small palpebral fissures, prominent nose, large ears and large flat mouth (Fig. 1a), as documented in cases 1 and 2. The neurological evaluation revealed mild postural hand tremor and normal saccadic eye movements. Visual and BAEP (Brainstem Auditory Evoked Potentials) evoked potentials were normal. The audiometric examination documented mild neurosensory hypoacusia. The EMG showed no sign of peripheral neuropathy and mild autonomic dysfunction. The MRI showed mega cisterna magna. At the cognitive and behavioural assessment, the patient was generally collaborating and cooperative. During the talks, she often showed propensity to give fast and impulsive answers. The evaluation by DSM-IV criteria revealed – as in patients 1 and 2 – the presence of impulsivity (compulsive buying disorder). Cognitive evaluation documented a borderline IQ of 78, with a light discrepancy among the verbal subtests and the performance ones (Verbal IQ = 81; Performance IQ = 76). As documented in the brother, she had good comprehension skills, but low attitudes in numerical reasoning and poor vocabulary. In addition, social insight, spatial perception, problem solving, logical and sequential reasoning appeared limited.

#### Patient 4

Patient 4 was a 16 years old girl, daughter of two non-consanguineous healthy parents. She was born at term after uneventful pregnancy (birth weight: 3,400 g, birth length: 50 cm). The mother had a spontaneous miscarriage during a previous pregnancy.

The clinical evaluation documented some dysmorphic features, such as low hair line and widely spaced nipples. In primary school she was noted to have learning difficulties associated with dyslexia and dyscalculia. She was diagnosed with a visual-praxis difficulty and a full-scale IQ at the lower end of normal range (77), with significant discrepancies between verbal (IQ = 100) and performance (IQ = 59) scores. At the neuropsychological evaluation the patient revealed low self-esteem, strong insecurity and poor social adaptation skills. Major depressive episodes, together with anxiety and distress, and behavioral disorganization were also well documented. Written communication skills were deeply impaired due to deficient visuospatial organization and global movement impairment. She started gaining weight progressively at the age of 6, and developed primary amenorrhoea at the age of 15. She presented thelarche and pubarche at the age of 13 and familial history of delayed puberty was reported in the paternal pedigree. Although GnRH stimulation test showed a pubertal response of LH and FSH (LH peak of 12.7 mU/ml, FSH peak of 11 mU/ml), with a concentration of oestradiol (E2) of 40 pg/ml (normal pubertal values >15 pg/ml), FSH and LH peaks were lower than the expected for age, according to functional immaturity of the hypothalamus-pituitary-gonadal axis. The brain MRI showed normal signal of both anterior and posterior pituitary gland, with a normal pituitary stalk. The MRI with contrast highlighted the presence of a microadenoma (35 mm) at the centre of the hypophysis.

#### Patient 5

Patient 5 was a 6 years old boy, son of two non-consanguineous healthy parents. His father suffered from idiopathic focal epilepsy of infancy and childhood, and the mother was healthy. He was born at term after an uneventful pregnancy (birth weight: 3,070 g, birth length: 50 cm). The proband also had a heterozygotic twin brother with normal neurological assessment, normal IQ score (88), and obesity.

A mild psychomotor delay was reported since the proband was only 5 months old. A delay of the expressive language was revealed at 2 years of life, with speech very poor and simple. Personal autonomy skills (e.g. toilet training, routine clothing, and use of cutlery) were also deeply impaired. At the present age the patient had bilateral iris and choroidal coloboma, Duane syndrome type 3 of the left eye (Fig. 1a), hypotonia of both arms and legs, and developmental coordination disorder (DCD), as confirmed by the Movement Assessment Battery for Children (MABC), with a score below the 5th percentile. The patient presented a borderline IQ [total IQ = 74, without discrepancy between verbal (IQ = 80) and performance (IQ = 82) scores] and required the support of a dedicated teacher. He also lacked proactivity in the relationship game and in accessing language expression, characterized by simple speech with a reduced vocabulary. The instrumental examination by Nuclear Magnetic Resonance (NMR) revealed hypoplasia of corpus callosum, inferior vermis and pons, as well as bilateral anomalies of the course of the sixth cranial nerve.

### Conventional and molecular karyotyping

In the three affected family members (patients 1–3), the karyotyping and the array-CGH analysis revealed a chromosome 21q interstitial deletion of approximately 10.6 Mb (chr21:21,754,822-32,380,347, hg38), and excluded the presence of any other genomic imbalance (Table 1, Fig. 1b and c). In patient 4, both karyotype and array-CGH detected a larger deletion, spanning approximately 14.5 Mb (chr21:13,048,294-27,532,614, hg38; Table 1, Fig. 1b and c). No other significant chromosomal rearrangements were revealed in the proband and the parental GTG-banded karyotypes showed a normal chromosomal asset. In case 5, the array-CGH demonstrated two genomic rearrangements: arr[hg38] 21q11.2q21.3 (14,000,146-27,785,985)x1 (Table 1, Fig. 1b and c), and the typical 220-kb deletion on chromosome 16p11.2 (OMIM 613444), arr[hg38] 16p11.2 (28,813,473-29,030,738)x1. The parental GTG-banded karyotypes were normal and the array-CGH analysis confirmed that the large 21q deletion appeared *de novo* in their child. On the contrary, the rearrangement on chromosome 16 was also detected in the mother and the proband’s heterozygotic twin. The molecular and clinical details of our cases have been referenced in the ClinVar database (http://www.ncbi.nlm.nih.gov/clinvar): Patients 1–3 (#SCV000239859), Patient 4 (#SCV000239860), Patient 5 (#SCV000239861).

**Table 1:** Summary of patients harboring 21q deletions overlapping with patients 1–5 and corresponding clinical features

Patient	Age at diagnosis (yr)	Phenotype (main features)	Chromosomal coordinates of deletion (hg38)	Size (Mb)	Genes (protein coding)	Inheritance	Pathogenicity
Patients 1-2-3 (present study)	53 (#1), 20 (#2), 18 (#3)	Obsessive-compulsive disorders, impaired social interactions, aggressiveness, delayed speech and language development, mild facial dismorphisms	chr21:21754822-32380347	10.63	112 (52)	Paternal	Pathogenic
Patient 4 (present study)	6	Intellectual disability, global movement impairment, dysmorphic features, dyslexia, dyscalculia, primary amenorrhoea, obesity, pituitary microadenoma	chr21:13048294-27532614	14.48	117 (21)	De novo	Pathogenic
Patient 5 (present study)	4	Intellectual disability, mild psychomotor delay, speech delay, hypotonia, DCD ^(a)^, Duane syndrome type 3, bilateral iris/choroidal coloboma	chr21:14000146-27785985	13.79	112 (23)	De novo	Pathogenic
			chr16:28813473-29030738	0.22	35 (24)	Maternal	Pathogenic
Case 1 (Petit et al., 2015) [24] Decipher#276325	7	Behavioural/psychiatric abnormality, attention deficit, impaired social interactions, frustration, aggressiveness, delayed speech and language development	chr21:21062316-24943120	3.88	16 (1)	Maternal	Unknown
Case 2 (Petit et al., 2015) [24] Decipher#254181	9	Global developmental delay, speech delay, hyperactivity, impairment of social interactions	chr21:15619936-23525918	7.91	50 (9)	Paternal (mosaicism)	Unknown
Case 3 (Petit et al., 2015) [24] Decipher#274603	5	Global developmental delay, hypotonia, constipation, impaired social interactions	chr21:16079383-24575840	8.50	48 (7)	Unknown	Unknown
KKI patient 3 - cohort A (Roberson et al., 2011)	6	Speech delay, mild/moderate mental retardation, dysmorfic features, hypotonia, GERD ^(b)^, eczema, dermatographism	chr21:16814345-33232252	16.42	159 (69)	De novo	Unknown
			chr4:65863868-66006319	0.14	0	Maternal	Unlikely pathogenic
			chr14:22625231–22795061 ^(c)^	0.17 ^(c)^	2 (2)	Paternal	Unlikely pathogenic
GM00137 - cohort B (Roberson et al., 2011) [1]	6	Severe psychomotor retardation, microcephaly, dysmorphic features, bilateral iris coloboma	chr21:13403408-28392024	14.99	124 (23)	Unknown	Unknown
			chr4:68917-11238519	11.17	229 (122)	Unknown	Unknown
GM06918 - cohort B (Roberson et al., 2011) [1]	9	Mental retardation, dysmorphic features	chr21:14981488-32298829	17.32	156 (61)	De novo	Unknown
Haldeman-Englert et al., 2010 [13]	2	Poor social interactions, speech delay, mild dysmorphic features, PDD-NOS ^(d)^	chr21:21085454-29813876	8.73	62 (18)	De novo	Pathogenic
Case 31 (Lyle et al., 2009) [2]	Unknown	Dysmorphic features, short stature, mental retardation, synbrachydactily	chr21:12965809-30890916	17.93	180 (56)	Unknown	Unknown
Case 32 (Lyle et al., 2009) [2]	Unknown	Dysmorphic features, short stature, mental retardation, microcephaly, clinodactily, hypotonia	chr21:12965809-30218169	17.26	145 (31)	Unknown	Unknown
Case 33 (Lyle et al., 2009) [2]	Unknown	Mental retardation	chr21:12965809-26199556	13.23	108 (15)	Unknown	Unknown
Hannachi et al., 2011 [20]	26	Moderate mental retardation, minor brain malformations, craniofacial dysmorphic features, azoospermia, diffuse cerebral atrophy	chr21:13603505-29194209	15.59	130 (23)	Maternal	Likely pathogenic
Decipher#285024	2	Ataxia, intellectual disability, poor speech, lower limb spasticity, speech articulation difficulties	chr21:13224687-27912651	14.69	124 (23)	Unknown	Pathogenic
Decipher#285691	10	Cognitive impairment, generalized myoclonic seizures, microcephaly, asymmetry of the ears	chr21:13045202-33522318	20.48	217 (76)	Unknown	Pathogenic
ECARUCA#4777	16	Mental retardation, seizures/abnormal EEG ^(e)^, short stature, prominent maxilla, dislocation of hip, atrial septum defect	chr21:14166659-20412272	6.25	66 (13)	De novo	Unknown
			chr21:43013575-46699983	3.69	116 (67)	De novo	Unknown
ECARUCA#4841	9	Mental retardation, seizures/abnormal EEG ^(e)^, facial dysmorphisms	chr21:15292766-19704615	4.41	31 (6)	De novo	Unknown

^(a)^ *DCD* developmental coordination disorder ^(b)^ *GERD* gastroesophageal reflux disease ^(c)^duplication ^(d)^ *PDD-NOS* pervasive developmental disorder not otherwise specified ^(e)^ *EEG* Electroencephalogram

## Discussion

Partial deletions of chromosome 21q are commonly associated with highly heterogeneous phenotypes. In this study we characterized five patients with partial 21q monosomies by array-CGH and conventional karyotyping. The three family members (patients 1–3) showed mild clinical features, such as facial dysmorphisms and behavioral abnormalities, mainly consisting of obsessive-compulsive features, poor social interactions and vulnerability to psychosis, fully expressed in the father. High levels of impulsivity, repeatedly identified as a major problem in schizophrenia, were present in all the family members: alcohol abuse (father), compulsive smoking (son) and shopping/spending addiction (daughter). The phenotypic intrafamilial variability might be due to additive genetic and environmental factors that potentially have accumulated in the oldest member of the family. A deletion comprising 21q21.2 and the proximal segment of 21q22.1 has been previously associated with schizophrenia susceptibility [6], although a recent multi-stage genome-wide association study failed to detect schizophrenia-associated genetic locus on chromosome 21 [7]. Interestingly, all family members presented postural hand tremor, a symptom never described in association with chromosome 21q deletions. In agreement with our observations, it has been reported that *Sod1−/−* mice presented tremors along with gait disturbances and skeletal muscle atrophy [8, 9]. However, according to Decipher, no cases of *SOD-1* deletions (chr21:31659622–31668930, hg38) have been associated with tremor until now and the unique family with *SOD-1* null mutation manifested an atypical form of familial amyotrophic lateral sclerosis [10].

**Fig. 2:**
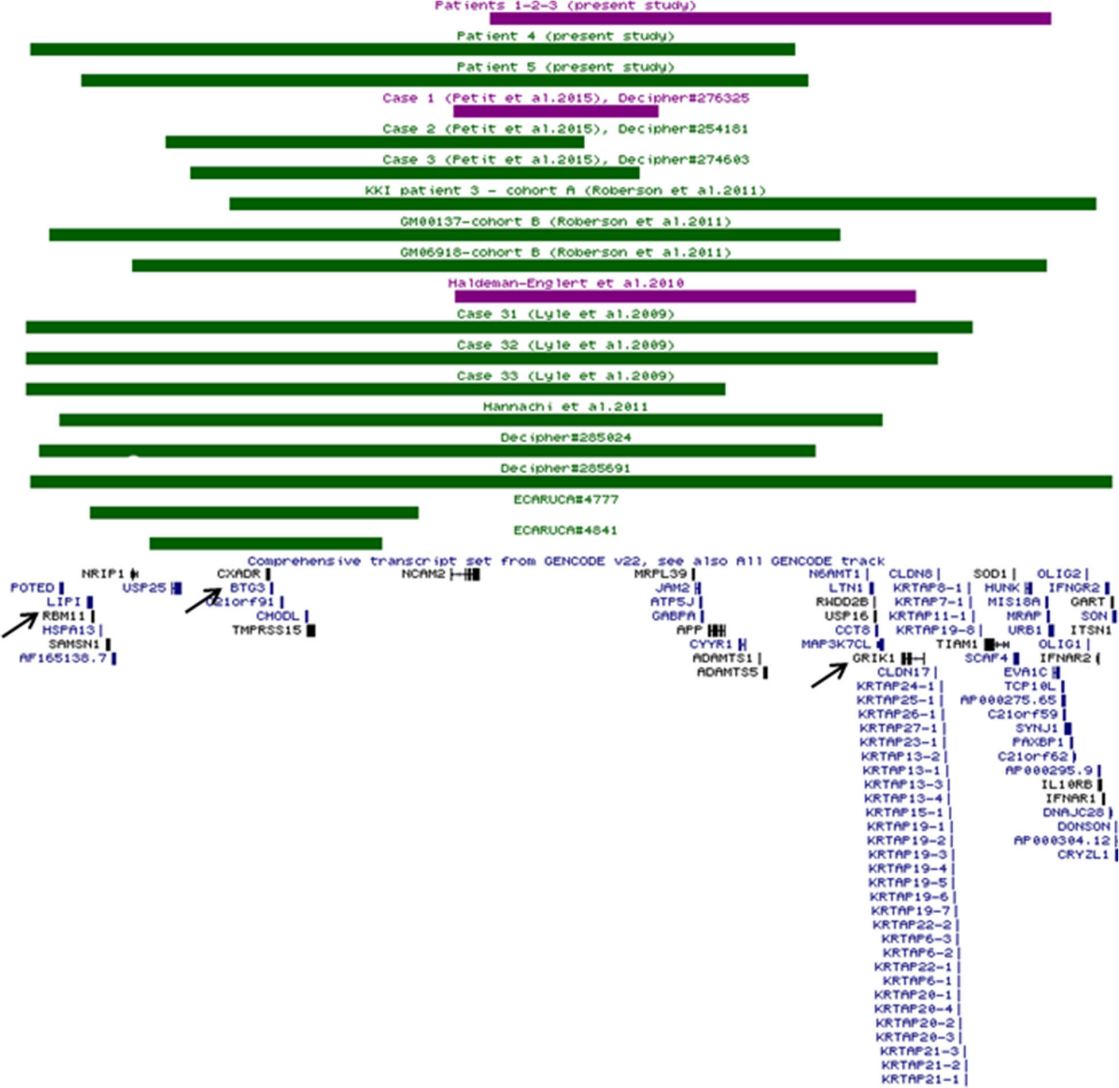
Comparison of 21q deletion cases with mild (purple) and moderate/severe (green) phenotypes (behavioral disorders and intellectual disability, respectively). The protein-coding genes of 21q region are mainly grouped into two main clusters. The proximal cluster includes genes more likely involved in intellectual disability (*BTG3* and *RBM11*), whereas the distal cluster mainly contains genes related to behavioral disorders, such as *GRIK1* (almost completely deleted in the case reported by Haldeman-Englert et al., [13]). KKI-3, GM00137, and ECARUCA#4777 cases also had rearrangements involving chromosomes other than 21 (as reported in Table 1) that might contribute to the clinical severity

Compared to patients 4 and 5, the three family members carried the deletion of *GRIK1* mapping to the 21q21.3 region (Fig. 2). This gene (OMIM 138245) might be considered a plausible candidate for autism and other neurobehavioral disorders, since it codifies for a protein belonging to the kainate family of excitatory glutamate receptors that are activated in a variety of neurophysiologic processes. Moreover, *GRIK1* alterations were shown to be associated with various neurobehavioral phenotypes in humans, such as anxiety disorders, schizophrenia, bipolar disorder, epilepsy and PDD-NOS (pervasive developmental disorder not otherwise specified) [11–13], as well as with anxiety-like behaviors in *GRIK1* knockout mice – due to its regulation of inhibitory circuits in the amygdala [14]. Accordingly, the behavioral disorders observed in our family suggest that *GRIK1* might be considered the most favorable candidate gene.

Patients 4 and 5, with similar proximal 21q deletions, showed the most severe clinical features, mainly consisting in intellectual disability (Table 1). Moreover, case 5 also harbored an additional deletion at 16p11.2, inherited from the clinically normal mother. Since this deletion has been previously linked to developmental delay, autism spectrum disorder and epilepsy [15–17], its additive effect on our patient’s phenotype might also be considered. In addition, the 220-kb 16p11.2 deletion has also been widely associated with susceptibility to isolated severe early-onset obesity (OMIM 613444) [18]. Interestingly, the proband and his mother were normal weight, whereas the proband’s heterozygotic twin harboring the same deletion was obese, thus supporting the incomplete penetrance and the clinical variability of this chromosomal alteration [19].

The findings that patients 4 and 5 presented more severe clinical features than patients 1–3 are in agreement with previously reported cases (Table 1 and Fig. 2) [1, 2, 20], where the proximal 21q deletions encompassed two genes expressed in the central nervous system, *RBM11* (21q11.2) and *BTG3* (21q21.1), that might play a role in intellectual disability. *RBM11* was deleted in 10 out of 15 cases with the most severe phenotype (intellectual disability), whereas *BTG3* in all of these cases (Fig. 2). *RBM11* is a tissue-specific splicing factor that mediates the alternative splicing process during neuronal differentiation [21]. *BTG3* (OMIM 605674) is involved in the neurogenesis of the developing central nervous system, where it acts as a regulator of cell proliferation and apoptosis [22]. Deletions of *BTG3* have been reported in a subset of patients with autism characterized by developmental regression [23] and in patients with neurodevelopmental delay [24] (Decipher 285691, 285987, 288573, 291626, and 300775). Moreover, *BTG3* deletions have also been associated with delayed speech (Decipher 249224, 277597, and 285024), as observed in our patient 5.

## Conclusions

**Fig. 3:**
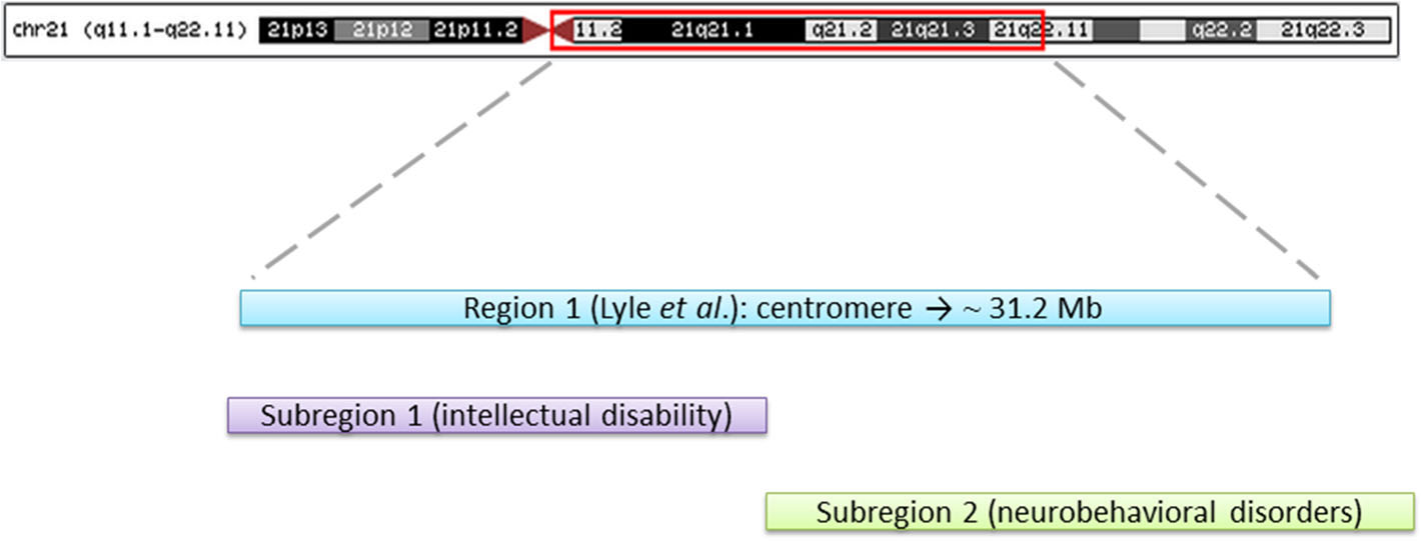
Subsetting of the great 21q region 1 described by Lyle and colleagues in 2009 into two smaller subregions. Deletions in the subregion 1, from the centromere to ~ 21 Mb (including *BTG3* and *RBM11*), are mainly associated with severe intellectual disability, whereas deletions of the subregion 2, until approximately 32 Mb (including *GRIK1*), are more tightly associated with milder neurobehavioral disorders, such as poor social interactions. Patients with a deletion overpassing the two subregions clinically manifested the most severe phenotype

Although further investigations of other cases are needed, our preliminary results provide new insights on the traditional model firstly proposed by Lyle and colleagues in 2009 [2], making it possible to tentatively subset their original great region 1 (21qcen-21q21.3) into two smaller subregions. Deletions in the subregion 1, spanning from the centromere to approximately 21 Mb (21q21.1), are mainly associated with intellectual disability, whereas deletions of subregion 2, until approximately 32 Mb (21q22.11), are more tightly associated with neurobehavioral disorders, such as obsessive-compulsive disorders, poor social interactions and vulnerability to psychosis (Fig. 3). Interestingly, the subregion 2 also includes a portion of the 21q22.11 band, whose deletion was traditionally considered associated with severe and even lethal phenotypes. This finding may be due to the fact that most of the disease-related genes, such as *SYNJ1*, *ITSN1*, *SLC5A3/SMIT1* and *KCNE2* [25–29], are clustered in the distal part of the band with the highest gene density.

According to the literature, very few cases of behavioral disorders with 21q deletions have been described until now [13, 24]. Indeed, attenuated phenotypes, such as poor social interactions, may be easily neglected and further genetic analyses are undertaken only when a suggestive familiar history is clearly ascertained. The spreading of genetic tests along with increasing evidences that copy number variations are linked to complex neuropsychiatric disorders [30, 31] will certainly unveil new cases in the near future.

## Methods

### Conventional karyotyping

Phytohaemagglutinin (PHA)-stimulated lymphocyte cultures were set up from peripheral blood samples and the chromosomal analysis was carried out on GTG banded metaphases, according to standard procedures.

### Molecular karyotyping

Molecular karyotyping (array-CGH) was performed on DNA samples, extracted from patient’s peripheral blood according to standard methods, by using a whole-genome 180 K Agilent array (Human Genome CGH Microarray, Agilent Technologies, Santa Clara, CA, USA), according to manufacturer’s protocol. Data were analyzed by using the Agilent Genomic Workbench Standard Edition 6.5.0.58. All genomic positions were reported according to the latest human genome assembly (GRCh38/hg38).

### Ethical approval and consent

The present study has been carried out according to the research rules of our institutional ethical committee on human experimentation and written informed consents were obtained from all the patients or their parents.

## Abbreviations

Array-CGH: array comparative genomic hybridization
BAEP: Brainstem Auditory Evoked Potentials
DCD: developmental coordination disorder
DSM-IV: diagnostic and statistical manual of mental disorders, fourth edition
EEG: electroencephalogram
EMG: electromyography
GERD: gastroesophageal reflux disease
IQ: intelligence quotient
MABC: movement assessment battery for children
MRI: magnetic resonance imaging
NMR: nuclear magnetic resonance
PDD-NOS: pervasive developmental disorder not otherwise specified
